# E-selectin ligand-1 controls circulating prostate cancer cell rolling/adhesion and metastasis

**DOI:** 10.18632/oncotarget.2503

**Published:** 2014-10-06

**Authors:** Sayeda Yasmin-Karim, Michael R. King, Edward M. Messing, Yi-Fen Lee

**Affiliations:** ^1^ Departments of Urology and Pathology and Laboratory Medicine, and Chemical Engineering, University of Rochester, Rochester, NY 14642; ^2^ Department of Biomedical Engineering, Cornell University, Ithaca, NY 14853

**Keywords:** prostate cancer, circulatory tumor cell, cancer cell rolling and adhesion, E-selectin ligand-1, E-selectin

## Abstract

Circulating prostate cancer (PCa) cells preferentially roll and adhere on bone marrow vascular endothelial cells, where abundant E-selectin and stromal cell-derived factor 1 (SDF-1) are expressed, subsequently initiating a cascade of activation events that eventually lead to the development of metastases. To elucidate the roles of circulating PCa cells' rolling and adhesion behaviors in cancer metastases, we applied a dynamic cylindrical flow-based microchannel device that is coated with E-selectin and SDF-1, mimicking capillary endothelium. Using this device we captured a small fraction of rolling PCa cells. These rolling cells display higher static adhesion ability, more aggressive cancer phenotypes and stem-like properties. Importantly, mice received rolling PCa cells, but not floating PCa cells, developed cancer metastases. Genes coding for E-selectin ligands and genes associated with cancer stem cells and metastasis were elevated in rolling PCa cells. Knock down of E-selectin ligand 1(ESL-1), significantly impaired PCa cells' rolling capacity and reduced cancer aggressiveness. Moreover, ESL-1 activates RAS and MAP kinase signal cascade, consequently inducing the downstream targets. In summary, circulating PCa cells' rolling capacity contributes to PCa metastasis, and that is in part controlled by ESL-1.

## INTRODUCTION

Metastasis is the most dreaded stage of cancer, accounting for the vast majority of prostate cancer (PCa)-related deaths [1–3]. Identifying the population at risk for developing advanced PCa and preventing metastases is one of the greatest challenges in cancer treatment today. Cancer metastases are the culmination of a complex series of steps where cancer cells degrade extracellular matrices allowing them to break away from the primary tumors. During this process, a subset of primary tumor cells undergo epithelial-mesenchymal transformation (EMT), involving morphogenetic changes to a mesenchymal cell-like phenotype of enhanced motility and plasticity that allows them to intravasate into the circulation and become circulating tumor cells (CTCs) [4–6]. Some CTCs can survive in the circulation, and eventually extravasate into distant metastatic locations [3], thus CTC numbers in PCa patients have been suggested as disease prognosis biomarker [7].

The process of extravasation involves several steps. First, CTCs roll on the endothelial cell surfaces which express E-selectin. This causes the CTCs to tether and roll in the post capillary venules [3, 8, 9]. This rolling process enables PCa cells to activate integrin signals, which trigger additional adhesion process, resulting in firm adhesions between cancer cells and endothelial cells [10]. This process promotes the transmigration of cancer cells through the endothelium to the metastatic sites [10, 11]. PCa cells preferentially metastasize to bone through site-specific interactions. Bone marrow endothelial cells express E-selectin, which enables homing of circulating PCa cells expressing E-selectin ligands to bone [12]. Not limited to PCa, the metastatic tumor cells from colon, breast, lung, ovary, and pancreas also have acquired the capacity to roll and adhere to the endothelial monolayer through E-selectin [13–16]. Our recent work has shown that alpha-1,3 fucosyltransferase 6, the key enzyme catalyzing E-selectin ligands, is an important regulator of PCa metastasis to bone [17]. It is reported that metastatic PCa tumors express elevated surface levels of E-selectin ligands, including CD 44, PSGL-1, ESL-1, beta-2-integrins and L-selectin [3, 9, 18–20]. ESL-1, one of E-selectin ligand, is a Ca^2+^ dependent inducible cell adhesion glycoprotein [21] and is encoded by a single gene locus named *Glg1* (Golgi-complex-localized glycoprotein-1), but its roles in cancer metastasis are not well known. In addition to E-selection, the stromal cell-derived factor 1 (SDF-1) and its receptor CXCR4 play a critical role in PCa bone metastasis. The CXCR4 positive PCa cells can form a firm adhesion to the osteocytes in the bone metastatic lesions that secrete/express SDF-1[22].

So far E-selectin has been recognized as the prime adhesion molecule expressed by the endothelium responsible for initiating rolling and adhesion of PCa cells [8], but there is a scarcity of knowledge about the role of rolling/adhesion of circulating PCa cells in terms of PCa aggressiveness/metastasis and the mechanism behind this. In this report we elucidate the roles of circulating PCa cells rolling and adhesion behavior in the development of metastatic PCa. To create a bone metastatic microenvironment of PCa we applied a dynamic flow-based E-selectin/SDF-1 coated microchannel system, mimicking bone marrow post capillary venules [23]. We demonstrated that circulating PCa cells' rolling/adhesion capacity contributes to PCa's distant metastasis, which is mediated via an E-selectin ligand, ESL-1. Consequently, the overexpression of ESL-1 transduces a cascade of signaling facilitating prostate cancer metastasis.

## RESULTS

### Circulating PCa cells' rolling capacity contributes to cancer aggressiveness

**Figure 1:**
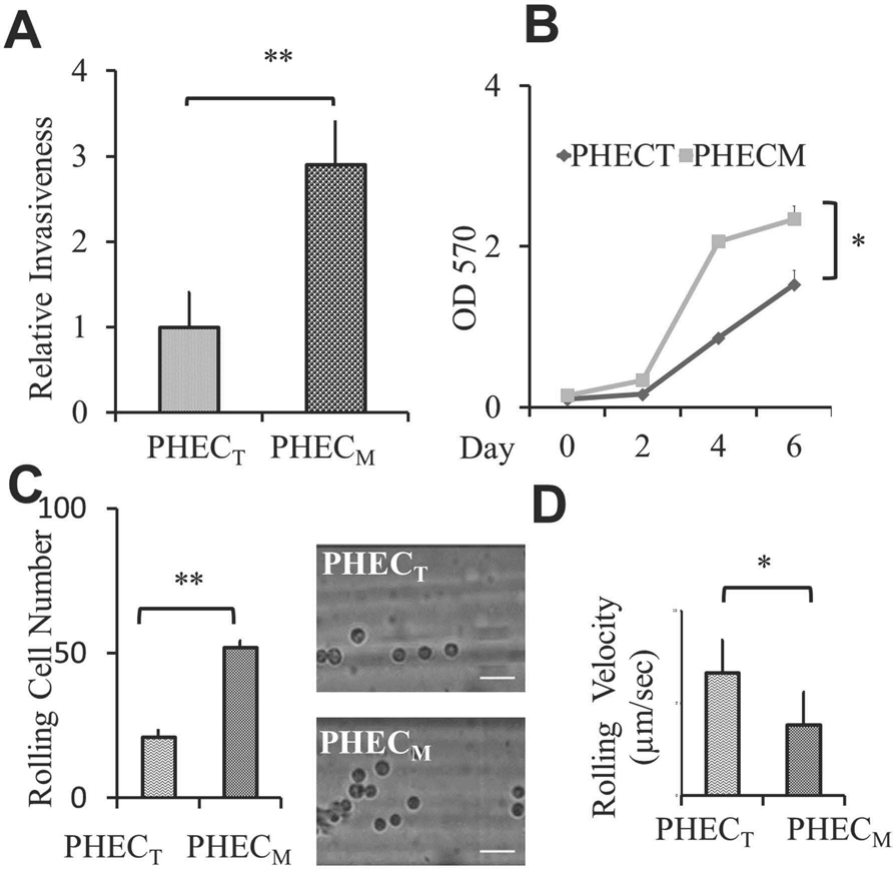
PCa cell aggressiveness is correlated with their rolling/adhesion capacity **(A)** Relative invasiveness of BPH-1derived PHEC_T_ and PHEC_M_ PCa cells. The Bar graph represents invasive cells per well using 24 well invasion chambers, the average of at least three experiments. **(B)** Line graph represents the MTT assay for cell proliferation. The results represent the average of at least three experiments. **(C)** Rolling cell number comparing the rolling behavior of BPH-1derived PHEC_T_ and PHEC_M_ cell lines using the microchannel system under wall shear stress of 1 dyne/cm^2^. Bar graph represents the average rolling/adhesion cell number of consecutive 10 frames of a video (200X) of one experiment. Pictures represent one frame. Scale bar represents 50 μm. **(D)** Rolling velocity of BPH-1derived PHEC_T_ and PHEC_M_ cell lines using the microchannel system under wall shear stress of 1 dyne/cm^2^. Graph represents the average rolling cell velocity of 10 cells of one experiment. Error bars indicate SEM. *, *P* < 0.05; **, *P* < 0.01.

To investigate if circulating PCa cells' rolling/adhesion behavior is an important PCa cell characteristic in the development of aggressive disease, we applied a dynamic flow-based system as illustrated in Supplementary Figure S1A and Supplementary Movie 1 [23]. First, we compared the rolling capacity among PCa cell lines with the same origin but different aggressiveness. Two BPH-1 derived cell lines that were originally established from hormone induced BPH-1 maliganant transformation in a tissue recombinant model were chosen [24]. These BPH-1 derived cell lines are PHEC_T_: isolated from primary tumors *vs* PHEC_M_: isolated from lymph node metastasis. As expected the metastatic PHEC_M_ cells demonstrated more aggressive cancer cellular phenotypes, higher invasiveness (Figure 1A) and higher growth rate (Figure 1B), as compared to the primary PHEC_T_ cell line. More importantly, PHEC_M_ displayed higher rolling cell numbers (Figure 1C) and lower rolling cell velocity (Figure 1D) as compared to primary tumor PHEC_T_ cells. This positive correlation of cancer aggressiveness with rolling capacity was further confirmed by two pairs of PCa cell lines; LNCaP-P *vs.* LNCaP-R and CWR22R-1 *vs.* CWR22R-2 [25, 26] where more aggressive PCa cells (Supplementary Figure S1B) also demonstrates higher rolling cell number (Supplementary Figure S1C) and lower rolling cell velocity (Supplementary Figure S1D) compared to their counterpart less aggressive PCa cells. Our data from three sets of PCa cell lines indicated that circulating PCa cells' rolling capacity is correlated with their aggressiveness and PCa rolling capacity is a novel cancer cell characteristic.

**Figure 2:**
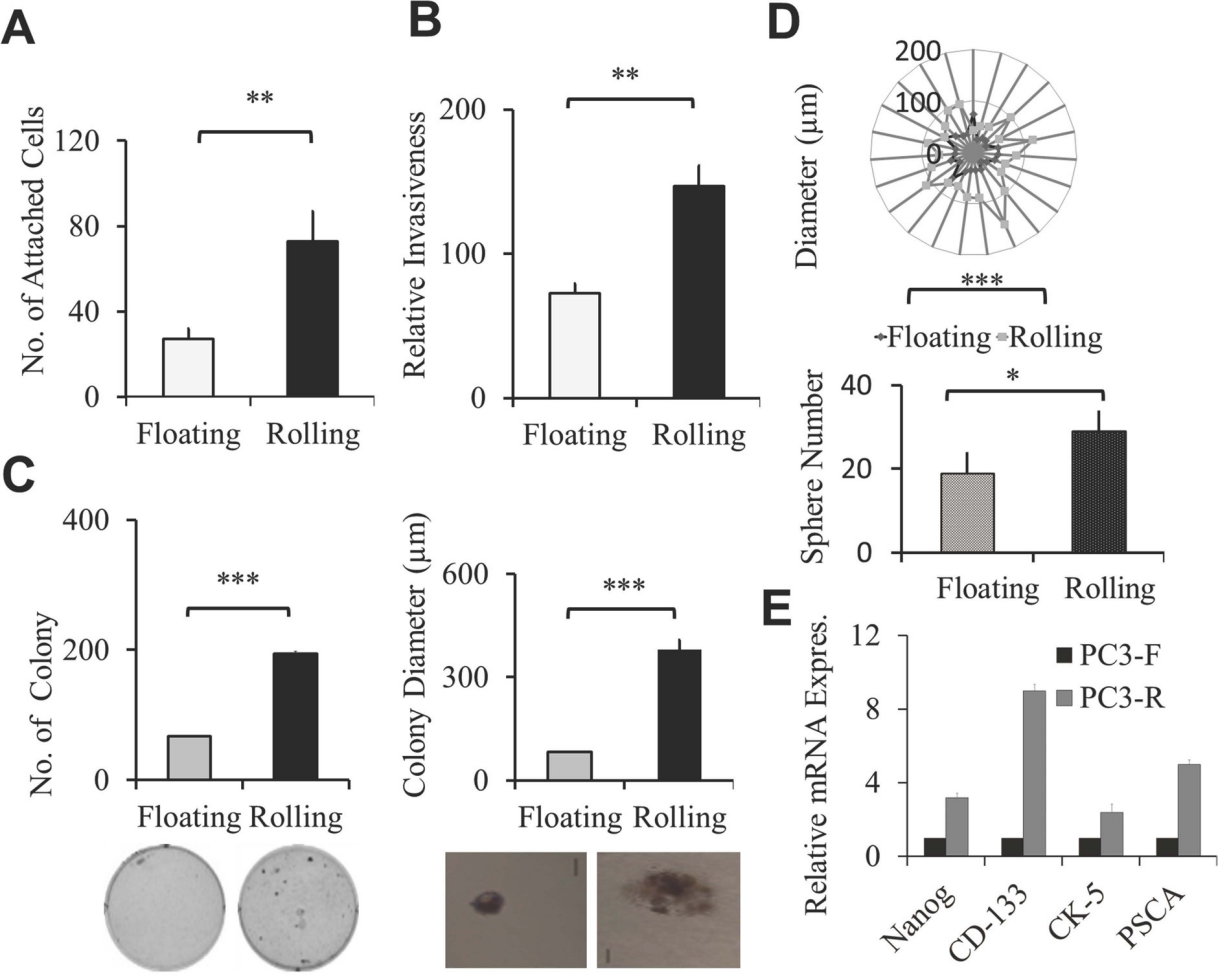
Sorted rolling PCa cells showed higher aggressive cellular cancer phenotype **(A)** Static adhesion assay of sorted rolling and floating PC-3 PCa cells on HUVEC cells. Calcein-AM stained rolling and floating PC-3 PCa cells were incubated on the monolayer of HUVEC cells. Adherent cells were counted under fluorescent microscope. Graphs represent the average adherent cell number per well. **(B)** Invasion assay of sorted rolling and floating PC-3 PCa cells. Bar graph represents relative invasiveness of rolling and floating cells/well in 24 well. **(C)** Anchorage independent growth by soft agar colony formation assay. Sorted rolling and floating PC-3 cells were seeded on soft agar gel and colony numbers were counted after 21 days. Bar graphs represent average number (left) and average diameter (right) of colony per well of 6 well plate. The pictures represent the size of one colony in 50x view. Scale bar 50 μm. **(D)** 3D sphere forming assay was used to determine the stemness of rolling and floating PC-3 PCa cells. Radar graph shows the diameter of spheres in μm, each dot represents one sphere. Bar graph represents the average sphere number per well. **(E)** Relative mRNA expression of stem cell markers in the sorted rolling and floating PC-3 cells was determined by QPCR. Results represent the average of at least 2 experiments. Error bars indicate SEM. *, *P* < 0.05; **, *P* < 0.01; ***, *P* < 0.001.

Circulating PCa cells are heterogeneous, and only a small percentage of circulating cancer cells can survive, roll and adhere, and eventually metastasize to a second target site. This flow-based microtube system provides a unique opportunity to isolate and characterize those aggressive circulating PCa cells that roll and adhere to microtubes. Therefore, we used this device to isolate PCa rolling population from two well-characterized metastatic PCa cell lines, PC-3 and DU145 cells. Cells infused through these coated microtubes under 1 dyne/cm^2^ shear stress were sorted and collected according to their adhesion ability (Supplementary Figure S1A). We found that more than 95% of PC-3 and DU145 cells were floating through the microtubes, defined as “floating” cells, and less than 5% of PCa cells roll and adhere to the microtubes, defined as “rolling” cells (Supplementary Movie 1). We then characterized these two sub-populations, rolling *vs* floating PCa cells, in different *in vitro* assays for their metastatic potential. First, we examined the sorted PCa adhesion ability to endothelial cells using the static adhesion assay in which PCa cells are allowed to settle on the endothelium. We found that both PC-3 and DU145 rolling cells have greater adherence to HUVEC cells compared to their corresponding floating cells in the static adhesion assay (Figure 2A and Supplementary Figure S2A). Next, we found that the rolling PC-3 as well as rolling DU145 cells invade more rapidly through the matrigel than corresponding floating cells (Figure 2B and Supplementary Figure S2B), and rolling PCa cells grow faster compare to the corresponding floating cells (Supplementary Figure S2C). Moreover, we performed the soft agar assay to measure the anchorage independent growth, one of the hallmark characteristics of cancer cells. We found that both rolling PC-3 and DU145 cells formed more and bigger colonies than floating PCa cells (Figure 2C and Supplementary Figure S2D). Finally, the cancer stem cell's characteristic which is associated with cell trafficking [27] and cancer aggressiveness [28] was examined. We compared the stemness between rolling and floating PCa cells by the sphere forming assay, and found rolling PCa cells developed more and larger sphere colonies in 3-D culture than the floating PCa cells (Figure 2D and Supplementary Figure S2E). Consistent with the cancer stem cell phenotype, we also found that the rolling PC-3 cells express higher levels of stem cell markers, such as NANOG, CD133, CK 5, and prostate specific stem cell marker (PSCA) [28, 29] compared to floating PC-3 cells by QPCR (Figure 2E). In summary, we successfully captured a small population of rolling PCa cells that represent the most aggressive circulating PCa cells using this flow-based device.

### Development of prostate metastatic tumors from rolling PC-3 cell implanted mice

**Figure 3:**
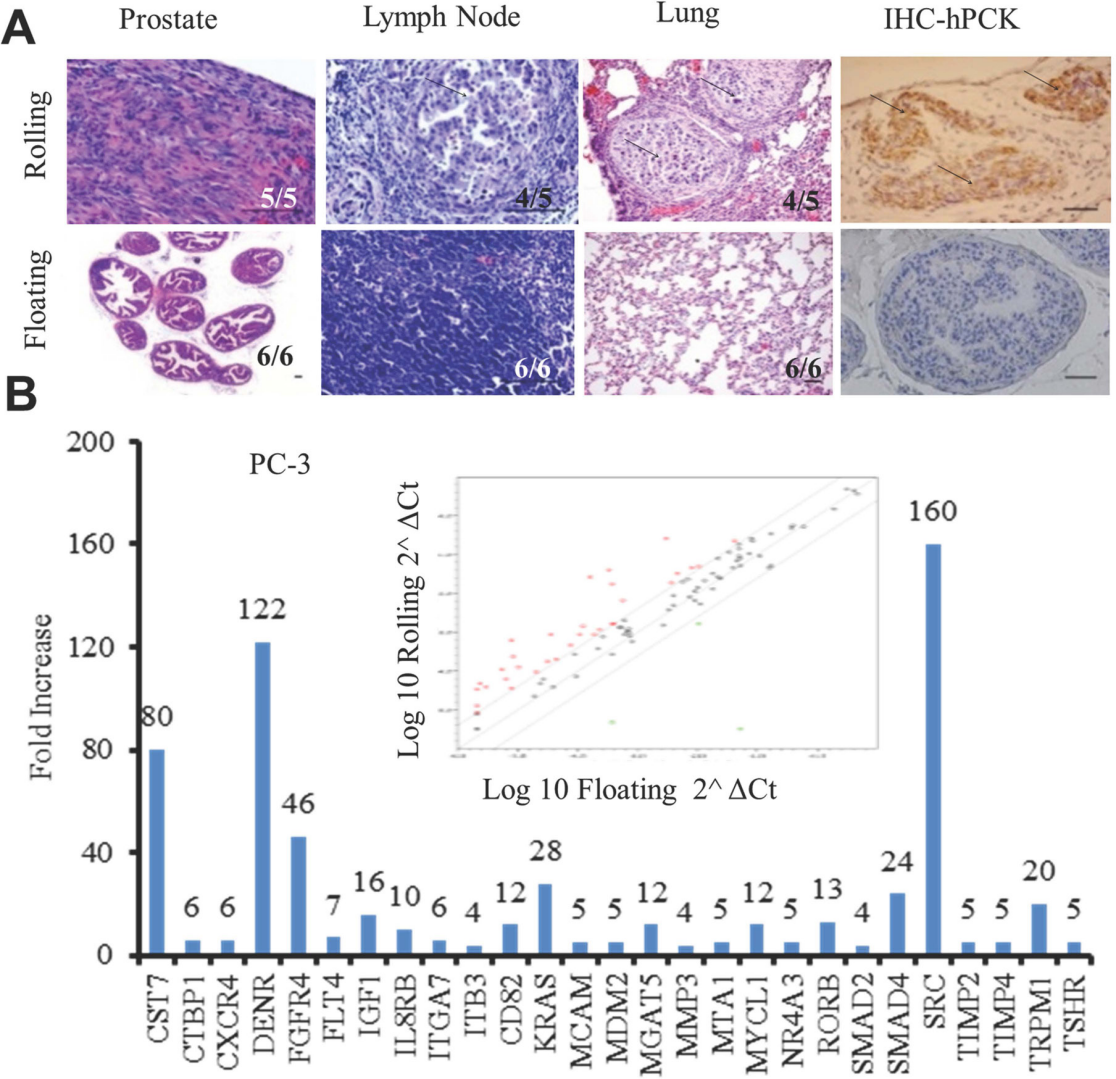
Rolling PCa cells demonstrate higher metastatic behavior *in vivo* and elevated metastatic related genes **(A**) Orthotopic xenograft mouse model was used to determine the metastatic potential of rolling and floating PCa cells *in vivo*. Mice were sacrificed three months after the implantation, and tissues were collected for histological examination. Histogram of first three columns showing the H&E staining of the rolling (upper row) and floating (lower row) mouse prostate tumors, lymph node and lung. The 4^th^ column represents the IHC staining of human pan cytokeratin in the rolling (upper) and floating (lower) prostate. Scale bar represents 50 μm. **(B)** Human metastatic gene array of 84 metastatic related genes. Scatter plot shows the up- and down-regulated genes (red dots for up and green dots for down) in sorted PC-3 cells and bar graph represents the upregulated metastatic genes (>2 fold) in rolling PC-3 cells, compared with floating.

To investigate rolling PCa's metastatic potential, we used an *in vivo* orthotopic xenograft mouse model. Freshly sorted rolling and floating PC-3 cells were implanted into the anterior prostate of B6 nude mice. Mice were sacrificed 90 days after tumor implantation and several targeted organs were harvested. We found that five of five mice implanted with rolling PC-3 cells showed large prostatic tumors, but strikingly all six mice implanted with floating PC-3 cells did not have any visible tumors within 90 days. Hematoxylin and eosin (H&E) staining showed normal glandular structures with intact basement membranes of the anterior prostate in all six floating PC-3 cell implanted mice (Figure 3A, first panel). More importantly, we found that four of five mice receiving rolling PC-3 cells developed metastatic tumors in their lymph nodes and lungs as shown in Figure 3A (second and third panel respectively). To confirm that the tumors are indeed derived from human PC-3 cells, we examined the expression of human Pan-cytokeratin (hPCK) by IHC staining and found hPCK positive staining only in the tumor tissues from mice receiving rolling PC-3 cells (Figure 3A, last panel). The development of metastatic tumors in the mice receiving rolling PCa cells strongly supports that the rolling PCa cells represent a subset of population in the circulation that may be responsible for cancer metastasis. The molecular alterations were further examined by the Human Metastasis Tumor Focused Gene Array (SABioscience) by comparing the differentially expressed genes between rolling and floating PCa cells. We found that 26 pro-metastatic genes in PC-3 rolling cells (Figure 3B) and 15 in DU145 rolling cells (Supplementary Figure S3A) were up-regulated in rolling PCa cells. We confirmed five of the common up-regulated genes in both, PC-3 and DU145 cells- IGF-1, MMP3, RORB, SMAD4, and TRPM1, as shown in a Venn diagram (Supplementary Figure S3B) by quantitative PCR using a second primer set (Supplementary Figure S3C). These data suggest that rolling PCa cells contain elevated metastatic related genes that may drive cancer metastasis.

### ESL-1 expression is elevated in rolling PCa cells and correlated with clinical PCa progression

**Figure 4:**
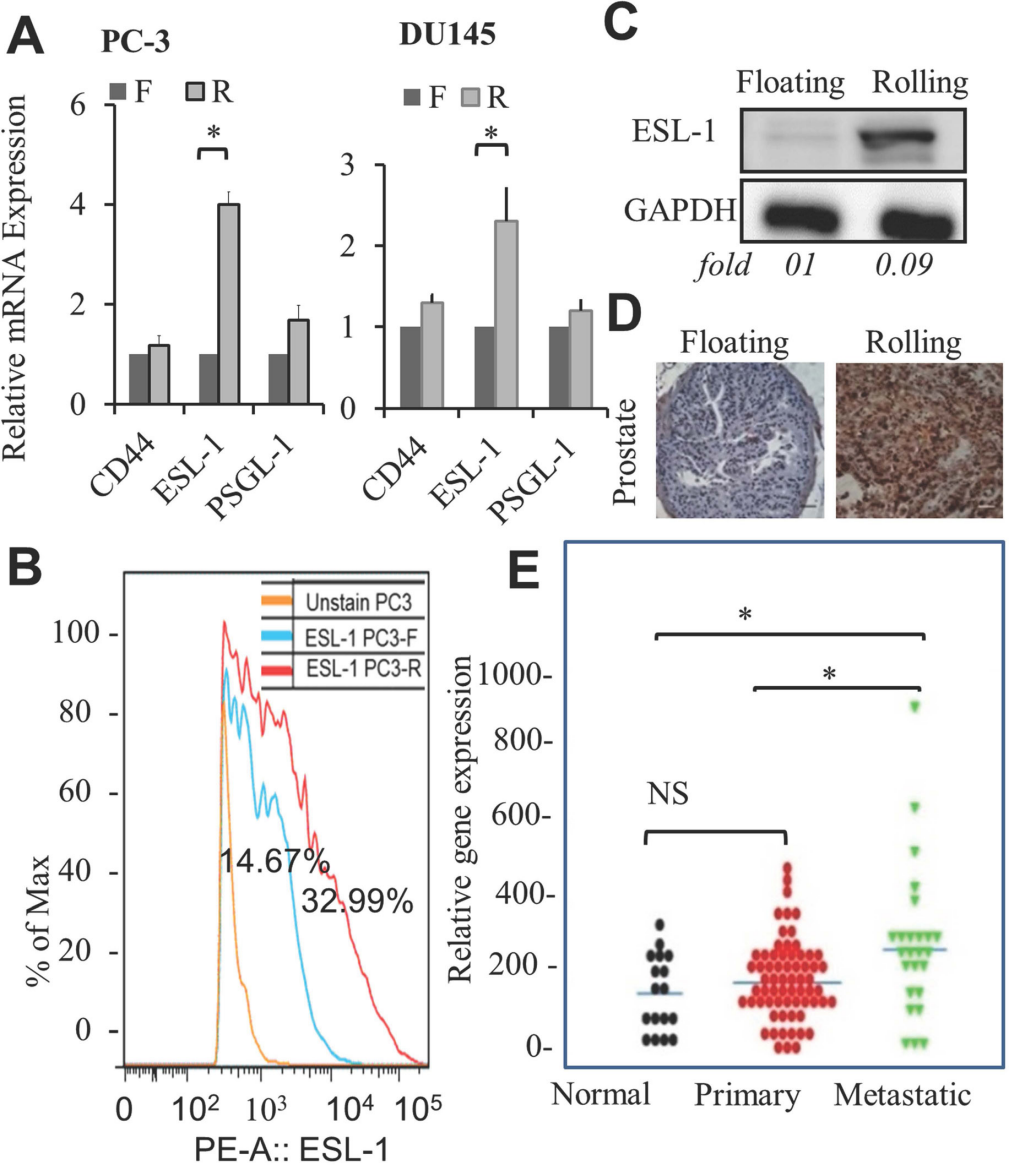
Elevation of ESL-1 in rolling PCa cells, tumors, and human metastatic tissue samples **(A)** Relative mRNA expression levels of E-selectin ligands; CD44, ESL-1 and PSGL-1 in sorted PC-3 and DU145 cells were examined by QPCR. Bar graph represents the average of 3 experiments. **(B)** Flowcytometry data showing surface expression of ESL-1 in sorted rolling and floating PC-3 PCa cells. Blue line represents PC-3 floating cells with surface ESL-1 expression on 14.67% and red line represents PC-3 rolling cells with surface ESL-1 expression on 32.99% cells. **(C)** Western blot analysis of ESL-1 expression in the prostate tissues of xenografted mice implanted with rolling and floating PC-3 cells. **(D)** ESL-1 expression in same prostate tissues by IHC staining with antibody to human ESL- 1. **(E)** The gene expression profile of ESL-1 was analyzed through public microarray repositories from NCBI Gene Expression Omnibus (Profile # GDS2546; Metastatic prostate cancer HG-U95B). Box plots represent expression level of ESL-1 in following 3 categories: normal prostate tissue free of any pathological alteration (n=18), primary PCa tumors (n=63) and distant metastasis samples (n=25). Error bars indicate SEM. *, *P* < 0.05.

When PCa cells come in contact with E-selectin coated surfaces, cells tether, roll, and ultimately adhere. The expression/profile of E-selectin ligands on PCa cells might be critical for such interaction that leads to PCa cells rolling or floating. Thus, we examined the expression levels of E-selectin ligands, CD44, ESL-1 and PSGL-1 on rolling and floating PCa cells. Among the ligands, we found that only ESL-1, but not CD44 or PSGL-1, showed a higher expression level in rolling PC-3 and DU145 cells in mRNA level (Figure 4A). Critically, using flow cytometry we confirmed that rolling PCa cells express elevated ESL-1 proteins on their surface (Figure 4B and Supplementary Figure S4A). More importantly, this elevated ESL-1 expression was also found in the xenografted prostate tumors derived from mice implanted with rolling PC-3 cells, as compared to prostate of mice receiving floating PC-3 cells by Western blotting analysis (Figure 4C) as well as immunohistochemistry of prostate tissues of floating and rolling PC-3 cell xenografted mice (Figure 4D). These data suggest that ESL-1 represents a novel PCa metastatic marker and its expression level is correlated with cancer aggressiveness. To confirm this, we examined ESL-1 expression between LNCaP sublines and found that elevated ESL-1 expression in the aggressive LNCaP subline (LNCaP-R) than that in less aggressive LNCaP-P subline (Figure Supplementary S4B). To access the clinical relevance of ESL-1, the ESL-expression in human PCa samples with different stages of disease were compared through public microarray repositories from NCBI Gene Expression Omnibus (Profile # GDS2546; metastatic prostate cancer HG-U95B). We compared the expression levels of ESL-1 in three groups: (1) normal prostate tissue (n=18), (2) primary PCa tumors (n=63) and (3) distant metastasis samples (n=25). We found high levels of ESL-1in metastatic tumors in comparison to normal prostate and primary tumors (Figure 4E), and no significant difference in normal prostate and primary PCa tumors. This elevated level of ESL-1 found in human PCa metastatic samples further supports ESL-1's roles in PCa metastasis; in particular, its status in circulating PCa cells could be an important functional biomarker for prediction of disease behavior.

### ESL-1 is responsible for PCa cell's rolling and adhesion

**Figure 5:**
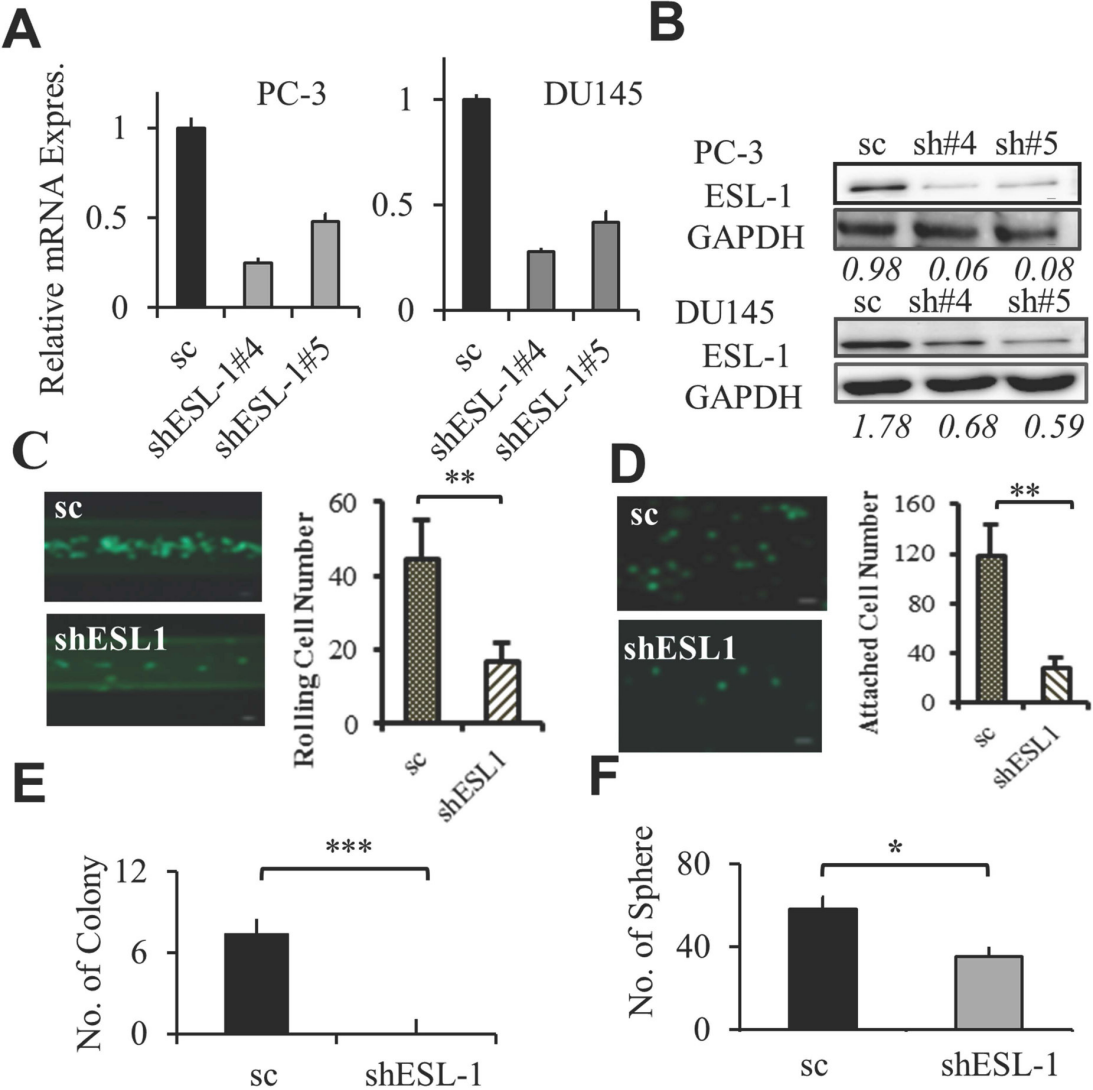
ESL-1 is responsible for PCa rolling and aggressiveness **(A)** Expression of ESL-1 in shESL-1 stable clones was determined by Q-PCR. Bar graph represents the fold reduction of ESL-1 expression in shESL-1clones compared to scramble control of PC-3 and DU145 cell lines. **(B)** Western blot analysis of ESL-1 expression in shESL-1clones compared to scramble control of PC-3 and DU145 cell lines. **(C)** Determining the rolling cell numbers of the shESL-1and scESL-1 PC-3 PCa cells using the microchannel system under flow of shear stress 1 dyne/cm^2^. Bar graph represents the average rolling/adhesion scESL-1 and shESL-1 PC-3 cell number of consecutive 10 frames of videos (100X) of one experiment. A representative picture is shown. Scale bar represents 50 μm. **(D)** Comparing the static adhesion between the shESL-1and scESL-1 PC-3 cells. Bar graphs represent the average adherent cell numbers of scESL-1 and shESL-1 PC-3 on HUVEC cells per well and the picture represents 50x view of the attached shESL-1and scESL-1 of PC-3 adherent cells. Scale bar represents 50 μm. **(E)** Anchorage independent soft agar colony formation assay with scESL-1and shESL-1 of PC-3 cell line. Colonies were counted after 28 days of culture. Bar graph represents the average colony numbers of three wells in 6 well plate (each were triplicated). **(F)** 3D sphere forming assay of scESL-1and shESL-1 of DU145 cell line. Bar graph represents average sphere numbers in three wells of 96 well plates. Results represent the average of at least 3 experiments. Error bars indicate SEM. *, *P* < 0.05; **, *P* < 0.01.

To determine if ESL-1 is a key factor that controls rolling, we knocked down ESL-1 expression in PC-3 and DU145 cells. Two shESL-1 (shESL-1#4 and shESL-1#5) and one scramble (scESL-1) stable cell lines were generated. The expression of ESL-1 was determined by QPCR and Western blot, and we showed that both shESL-1#4 and #5 ESL-1 clones express significantly lower level of ESL-1 than scESL-1(Figure 5A and B). Those shESL-1 cells were then subjected into *in vitro* assays to test their rolling capacity and aggressiveness. As expected, knocking down ESL-1 impairs PCa cells' rolling capacity significantly, where we found a significantly fewer number of shESL-1 PC-3 and shESL-1 DU145 cells can roll and adhere to the coated microtubes under shear stress 1 dyne/cm^2^ (Figure 5C and Supplementary Figure S5A). In addition, shESL-1 PCa cells displayed significantly less static adhesion ability than scESL-1cells to the endothelial cells (Figure 5D and Supplementary Figure S5B). Strikingly, the colony formation ability was significantly impaired when ESL-1 was knocked down where shESL-1 PC-3 cells did not develop any significant colony in 28 days of culture (Figure 5E). Interestingly, knocking down ESL-1 also impairs stem cells activity where shESL-1 DU145 cells had reduced number of spheres compared to scESL-1 cells (Figure 5F). In summary, these data support a critical role of ESL-1 not only in controlling cancer cell rolling/adhesion ability, but also in the tumorigenicity, both of which are important for cancer metastasis.

### ESL-1 activates RAS-MAP Kinase signaling pathway

**Figure 6:**
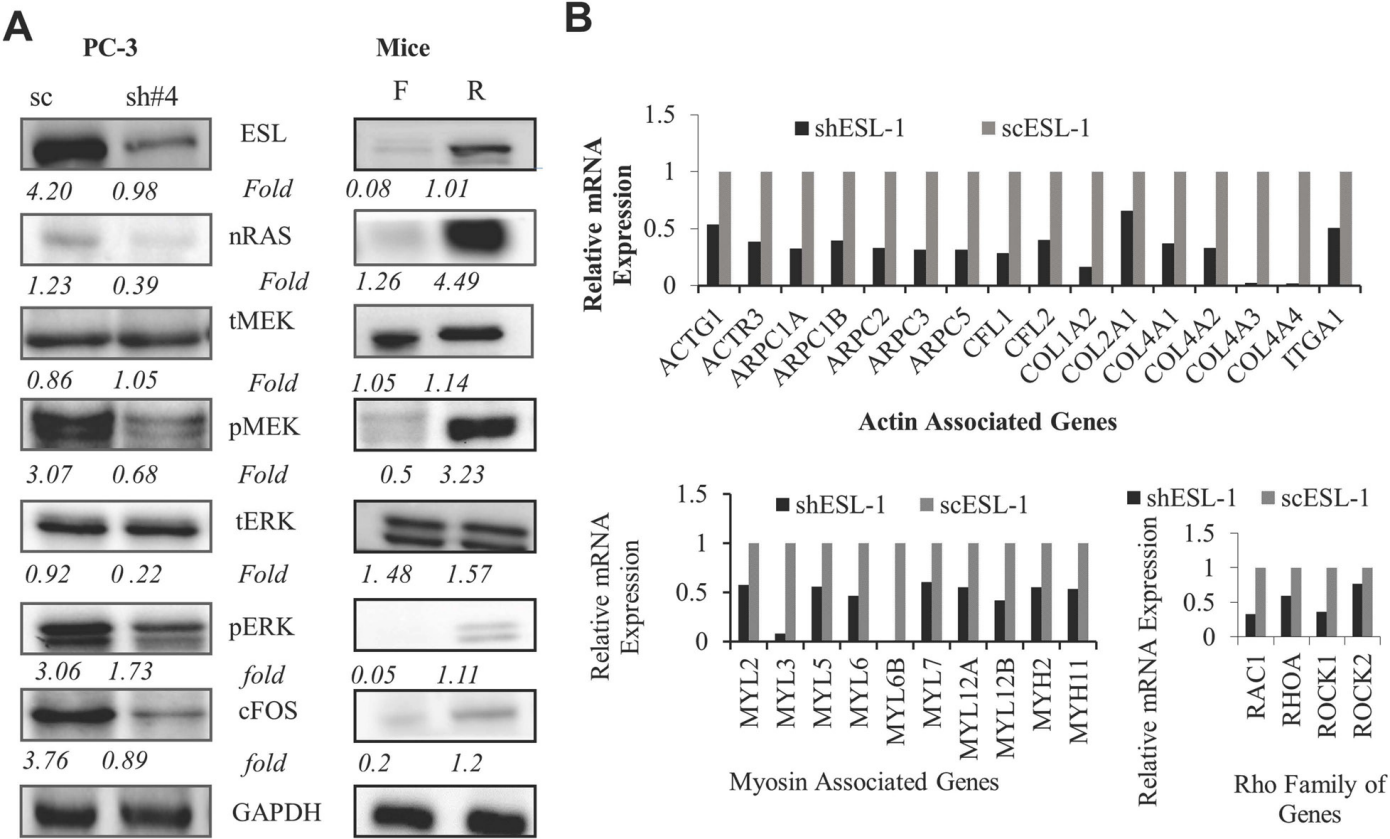
ESL-1 activates RAS-MAP kinase signaling pathway **(A)** Western blot analysis of key molecules of RAS-MAP kinase signaling pathway from cell lysate of shESL-1 and scESL-1 of PC-3 cells (left panel) and prostate tissues from the orthotopic mice receiving rolling and floating PC-3 cells (right panel). They are ESL-1, nRAS, total MEK, phosphoMEK, total ERK1/2, phosphoERK1/2, and its downstream transcription factor, cFOS antibody. GAPDH was used as a housekeeping gene. **(B)** Integrin-mediated cell adhesion and migration PCR array. Cell adhesion and migration PCR array was performed to compare the cytoskeleton gene expression in scESL-1 and shESL-1 PC-3 cell line. Bar graph represents the relative fold expression of 3 clusters of genes representing Actin, Myosin, and Rho family.

The activation of MAP kinase pathways has been associated with cell migration and invasion [30]. Of particular importance, activation of MAP kinase is associated with cancer metastasis, and elevated levels of phosphorylation of key signals such as ERK1/2 are often found in invasive cancer. To uncover if ESL-1could promote the Ras induced oncogenic pathway, we performed the Western blot and found that knocking down ESL-1 decreased the expression of nRAS, phosphoMEK, phosphoERK1/2 and its downstream transcription factor, cFos (Figure 6A left panel and Supplementary Figure S6). Critically, the elevated expression levels of MAP kinase signal molecules, such as nRAS, pMEK, pERK1/2 and cFOS were found in the prostate tumors derived from the xenografted mice who received high ESL-1 rolling PC-3 cells (Figure 6A. right panel). These data implied that PCa cells express high levels of ESL-1, which may be responsible for the activation of pMEK and pERK mediated cascade of signaling pathway, consequently enhancing their potential to invade and grow in a second target organ.

To further reveal the ESL-1 downstream target genes in controlling cancer metastasis, we applied the integrin-mediated cell adhesion and migration PCR array (Bio-Rad), because selectins and their ligands orchestrate the cell mobility/adhesion mainly through an integrin dependent mechanism by activating different proteins for cytoskeletal remodeling [31]. As shown in Figure 6B, we found that a number of actin and myosin related genes including Rho family were down-regulated in the shESL-1 cells. Rho GTPases regulate actin polymerization and myosin activity during adhesion dynamics [31]. Our data suggested that ESL-1 is the key factor in circulating PCa cells' rolling/adhesion behavior.

**Figure 7:**
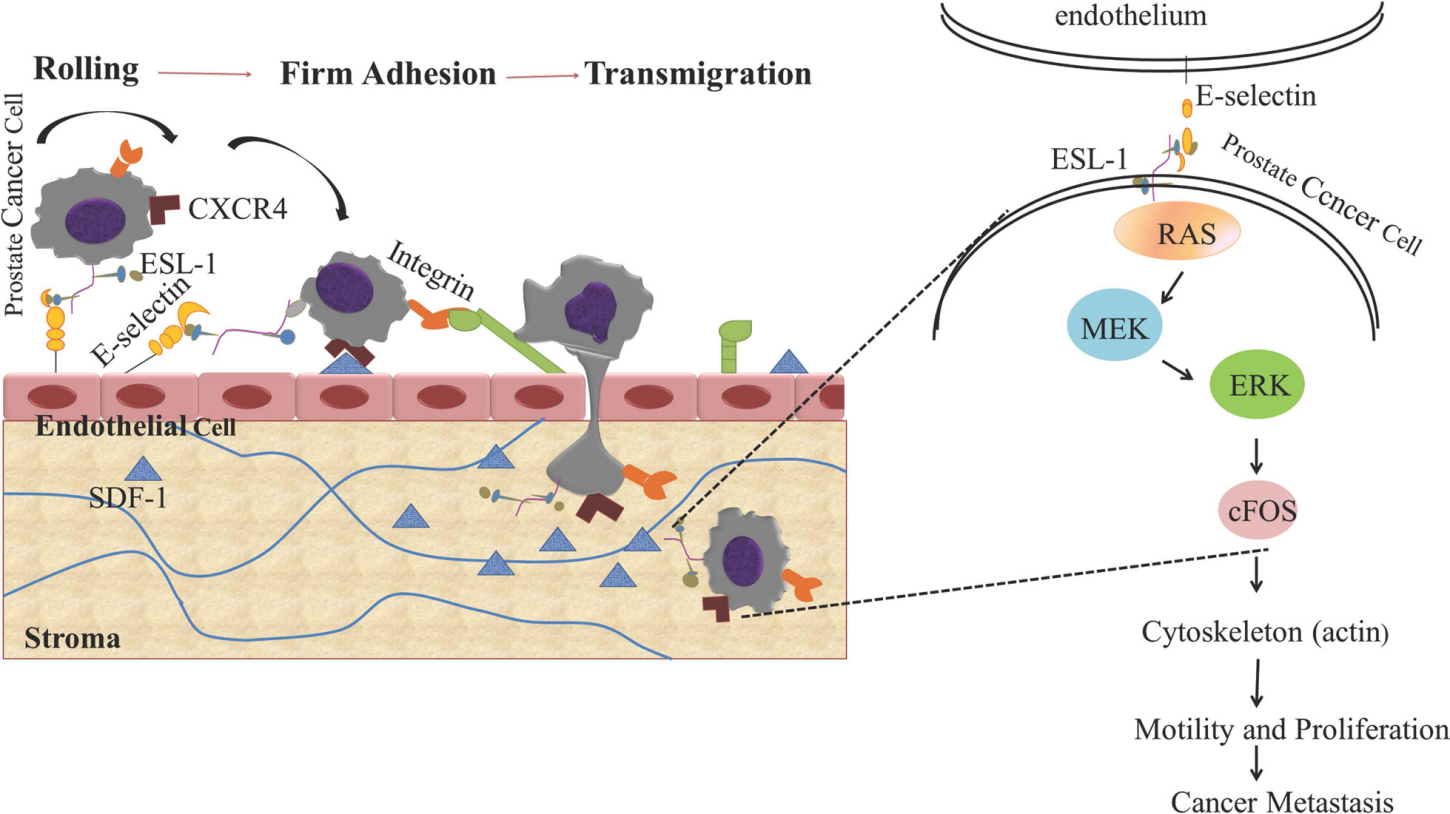
Schematic diagram of the oncogenic signaling pathways induced by E-selectin/ESL-1- mediated PCa rolling and adhesion on endothelial cell surface PCa cells express high level of E-selectin ligand, ESL-1, which mediates the rolling/adhesion of PCa cells on endothelial surface through the RAS-ERK- MAP kinase pathway.

In summary, circulating PCa cells expressing high level of ESL-1 can be stalled and roll on the endothelial cells where E-selectin is expressed. Moreover, we identify ESL-1, as a novel marker for PCa metastasis via controlling cancer cell rolling/adhesion. Overexpression of ESL-1 in prostate cancer cells triggers a signaling kinase cascade, from activation of RAS, MEK, and ERK, consequently inducing the expression transcription factor, and activates a cascade of gene expressions that allows cancer cells to migrate, invade and spread to the secondary organs (Figure 7).

## DISCUSSION

During cancer metastasis, CTCs follow a selectin based migratory pathway that is similar to leukocytes during the inflammatory response [4, 6, 21, 32, 33]. Dimitroff et al have shown that E-selectin ligands, such as PSGL-1, are over-expressed in human prostate bone metastasis tissues [8], suggesting a critical role for E-selectin ligands in cancer metastasis. To date, few studies have reported on the correlation between expression of the E-selectin ligands and prostate cancer bone metastasis [9, 12, 18, 34]. In this study, we identified ESL-1 as the key factor that controls PCa cells' rolling capacity under the flow, and knocking down ESL-1 impairs PCa cell rolling and mobility, and disrupts anchorage independent growth. This is the first study that demonstrates the functional roles of ESL-1 in controlling PCa rolling/adhesion and metastasis. ESL-1 is a Ca^2+^ dependent inducible cell adhesion glycoprotein, responsible for converting the initial tethering into slow rolling in myeloid cells [35]. It is also believed to be a variant of the receptor of fibroblast growth factor (FGF) [36, 37], which may demonstrates the interesting relationship between the high expression of ESL-1 with PCa growth rate, as FGF is known to be one of the growth factors in cancer progression [38].

The ESL-l mediated pathways that control cancer cell adhesion/rolling and metastasis are largely unknown. In this report, we showed that overexpression of ESL-1 activates Ras-MAP kinase cascade in PCa cells, then triggers the expression of downstream targets, like integrin mediated cytoskeleton system (action and myosin polymerization) and the Rho family of proteins [31]. The ERK signaling pathway is known to control diverse cellular processes, and was found to be often up-regulated in human tumors. ERK signal has been found to be important for the osteomimetic properties of the bone metastasis of prostate cancer cell lines [39], and expression of the key signal factors such as MEK5 is found to be overexpressed in PCa metastatic tissues [40] and can promote PCa cell mobility. Moreover, we also identified ESL-1 downstream targeted genes that are involved in the cytoskeleton movement. All of these proteins are required for cell mobility, cell migration, and cell adhesion and all those cellular behaviors are essential for cancer progression during distant metastasis. In summary, our study reported a novel pathway that controls cell rolling and adhesion, from ESL-1 to RAS-ERK, then to cytoskeleton proteins. Targeting ESL-1 and/or its regulatory signal molecules, and/or downstream oncogenic proteins may provide a novel therapeutic strategy, especially for those patients who have a high ESL-1 expression in CTCs.

Our findings on ESL-1's oncogenic properties and its elevated expression profile in clinical metastases are clinically significant. First, ESL-1 can be used as a novel functional biomarker for CTCs that can be used to predict patients' outcomes, i.e., to include not only patients' CTCs numbers, but also the expression level of ESL-1 to determine CTCs' “bioactivity”. More sensitive assays, such as an immunofluorescence assay, and an in-depth large scale clinical study are required to further establish the correlation of ESL-1 in CTCs with patients' outcomes. Second, this identified oncogenic pathway(s) induced by ESL-1 can serve as a principle for developing targeted therapy for those patients who have higher levels of ESL-1 in their CTCs.

Metastasis is the most dreaded stage of cancer and a major cause of death from prostate cancer. Therefore, understanding the PCa metastatic process, and identifying the essential molecular events during metastases are important tasks. Our study provides a model system to isolate and characterize a small rolling population of CTCs, and identifies a key molecule, ESL-1, for its roles in CTCs rolling and cancer metastasis. Further clinical evaluation of the expression levels of ESL-1 in patients and corresponding clinical outcomes are highly anticipated, and better therapeutics designed to block CTCs rolling and adhesion require further investigation.

## MATERIALS AND METHODS

### Cell Lines, molecules, and antibodies

BPH-1 series: PHEC_T_ and PHEC_M_ human prostate epithelial BPH-1 series of cells were provided by Dr. William Ricke. BPH-1 cells undergo malignant transformation when they are recombined with mouse urogenical mesenchymal cells (mUGM) under the kidney capsule. PHEC_T_ was derived from non-metastatic primary tumor and PHEC_M_ was derived from lymph node metastasis [24]. LNCaP-P and LNCaP-R, were established and CWR22R-1 and CWR22R-2 were tested in our laboratory where LNCaP-R and CWR22R-2 cells are resistant to chemotherapy drugs such as vitamin D and exhibit more aggressive behavior as compared to their parental line (LNCaP-P and CWR22R-1) [25, 26]. PC-3 and DU145 cell lines were obtained from American Type Culture Collection (ATCC). Primary human umbilical vein endothelial cells (HUVEC) were a generous gift from the Aab Cardiovascular Research Institute of the University of Rochester. PHEC_T_ and PHEC_M,_ LNCaP-P, LNCaP-R, CWR22R-1, CWR22R-2, PC-3, and DU145 cell lines were cultured with RPMI-1640 medium (GIBCO) containing 10% fetal bovine serum and 1% streptomycin/penicillin. Primary Human Umbilical Vein Endothelial Cells (HUVEC) were maintained in Medium 200 (Gibco, Carlsbad, CA), containing 1x low LSGS and 2% heat-inactivated fetal bovine serum [41]. Recombinant human E-selectin and SDF-1β were purchased from R&D systems.

### Preparation of microtube for cell rolling and capture

The inner surface of the blood vessel-compatible Micro-Renathane tube (MRE 025, inside diameter 300 μm, length 50 cm, Braintree Scientific Inc.) was coated with recombinant human E-selectin/Fc chimera (rhE/Fc, R&D Systems), and SDF-1β (R&D Systems) at 40 μg/ml and 10 μg/ml concentrations respectively in Dulbecco's Phosphate Buffered saline (DPBS, GIBCO) by incubating two hours at room temperature. Then, the microtubes were gently and slowly washed with calcium-enriched Hanks balanced salt solution (HBSS^+^, pH 7.4, 2 mM Ca^++^, GIBCO) [23] and after 15 minutes were ready for rolling assay.

### PCa cell rolling on microtube

PCa cells were detached from the culture dish surface by cell dissociation buffer (GIBCO) for 10 minutes, and washed with cell culture medium. Cells were suspended in Ca^++^ HBSS buffer for 15 to 30 minutes on ice. Cell numbers and viability were determined, and cell density was controlled at 1-2×10^5^cells/ml. MRE tubes were positioned on the stage of an Olympus IX-81 microscope coupled to a Hitachi CCD camera KP-M1AN (Hitachi, Tokyo, Japan) for direct visualization of the cells in the lumen [23]. PCa cells were infused through the tubes at 1 dyne/cm^2^ wall shear stress using a syringe pump (New Era) (Supplementary Figure S1). The images were captured using StreamPix and data were analyzed.

### Determining rolling cell number and rolling velocity

Rolling PCa cells were defined as cells translating at < 50% of the calculated hydrodynamic free stream velocity and cells that remained stationary for more than 10 seconds or rolled less than four cell diameters were considered as rolling cell [42]. The rolling cell number was determined as the average cells captured from 10 consecutive frames of the film captured at 200X. Image J and Excel software were used to measure the change in position of the cell center in a given period of time to calculate the rolling velocity. The time and distance of travel of 10 random rolling cells from each one minute of film was calculated. Rolling velocity was calculated [23].

### Sorting PCa cells

To sort PCa cells into rolling and floating subpopulations, PC-3 and DU145 cells were prepared [43] and infused through coated MRE tubes at a physiological capillary shear stress of 1 dyne/cm^2^ [44]. After 30 minutes of infusion, each tube was infused with Ca^++^ HBSS buffer to wash out all of the unbound cells. The unattached (named as “floating”) cells were collected at the other end of the tube. Cells adhering to the tube (named as “rolling”) were collected by using a high shear with calcium free DPBS. Cell number count and viability of the collected cells were analyzed using a Vi-Cell XR Cell Viability AnalyZer (Beckman Coulter) and >85% viable cells were used for experiments. Also to reduce the heterogeneity of the cells during cell culture, only freshly sorted cells were used in all of the experiments.

### Static adhesion assay

HUVEC cells were grown as a monolayer in a 6-well plate to obtain confluence up to 90%. Freshly sorted “floating” and “rolling” PCa cells were labeled with GFP by incubating with 1 μM Calcein-AM fluorescent dye (Sigma) for 30 minutes at 37°C and washed with PBS. Floating and rolling Calcein-AM labeled PCa cells were seeded on the monolayer of HUVEC cells (5×10^3^/well) and incubated for 30 minutes at 37°C with 5% CO_2_. Unbound PCa cells were washed out with PBS. Adherent cells were counted and photographs were taken using a Zeiss Axio A1 microscope coupled with Zenoptik camera measuring the intensity of fluorescent at 494/517 nm (Abs/Em) [23]. For shESL-1 and scESL-1 PCa cells, no Calcein-AM labeling was required since both cells express GFP.

### Sphere forming assay

Single cell suspension was seeded at a density of 100 cells/well in 96 well plates on the top of a thin layer of matrigel (BD Bioscience) [45]. RPMI cell culture media with 10% FBS and 4% matrigel was added on the top every other day. Cells were cultured up to 8 days. Colonies were counted and photographs were taken using a Zeiss Axio A1 microscope coupled with Zenoptik camera.

### Colony forming assay

In an anchorage-independent colony forming assay, sorted PCa cells were suspended at a density of 10^3^ cells/well in 0.4% low melting point agarose in RPMI cell culture media, and plated on top of 2 ml underlayer of 0.8% agarose in the same medium, in 6-well culture plates. Medium was refreshed twice per week for three weeks, stained with p-iodonitrotetrazolium violet and colonies containing more than 50 cells were counted and photographed with a Zeiss Axio A1 microscope coupled with Zenoptik camera. Diameter was measured from the photograph using Image J software [26, 46].

### Flow cytometry

Expression of ESL-1 was analyzed by Flow cytometry (BD FACS Canto II) following standard protocol [47] and was analyze using FlowJo software. Anti ESL-1 antibody (ab58826 from ABCam) and PE labeled secondary antibody was used from Miltenyi Biotech following the manufacturer's protocol. To detect the total ESL-1, cells were permeabilized with 0.1% PBS-Tween for 20 minutes. Cells were prepared following the manufacturer's instruction.

### Human metastatic and adhesion/migration gene array

To compare the gene profile alteration on the two sorted subpopulations of PCa cells, tumor metastasis focus using RT2 Profiler PCR Array of Human Tumor Metastasis (SABioscience) kit was used to do metastatic gene array to determine and compare up and down regulated metastatic gene among sorted “floating” and “rolling” PCa cell population. In addition, we also applied Integrin Mediated Cell Adhesion and Migration H96 predesigned 96 well array panel (BioRad).

### Orthotopic xenograft

1×10^6^ sorted, viable (>85%), PC-3 floating and rolling cells suspended in BD Matrigel were implanted to the anterior prostate of 10 week old nude mice (6 for floating and 5 for rolling) [22]. The mice were euthanized after 90 days, the prostate, adjacent lymph nodes, liver, and lungs were collected for histological analysis, and targeted proteins expression was measured by Western blotting analysis. Photographs were taken using a Cannon digital camera and histological photographs were taken using a Zeiss Axio A1 microscope coupled with Zenoptik camera and Leica DM5000 B microscope coupled with Optronics MacroFire digital camera.

### Immunohistochemistry (IHC) staining

IHC staining was performed following the standard protocol [48]. The sections were stained with the primary antibody before being analyzed with the commercial kit. The sections were counterstained with Hematoxylin (Ricca Chemical Company Catalogue No. 3530-16) and rinsed with tap water. Sections were observed and the micrographs were pictured using Leica DM5000 B microscope coupled with Optronics MacroFire digital camera. Antibodies for IHC: Cocktail of Human Pancytokeratin was used to identify human cytokeratin in the human PC-3 cell implanted metastatic cancers. Dilution was 1:200. Mixture of 2 antibodies was used: BD Bioscience, antihuman-cytokeratin, CAM 5.2 and Dako, monoclonal mouse anti-human cytokeratin, #M3515, clone: AE1/AE3. For staining ESL-1 anti ESL-1 antibody (Abcam, Cat # ab103439) was used in prostate tissues.

### Establishment of shESL-1 PCa cells

ESL-1 shRNA and universal scramble control plasmids were constructed in lentiviral pGIPZ vector (Open Biosystems) following the company protocol. To establish the stable cells we transfected pGIPZ-scramble or ESL-1 shRNA (pGIPZ-ESL-1 shRNA) with pGIPZ - psPAX2 (virus-packaging plasmid), and pMD_2_G (envelope plasmid) into 293 cells using Lipofectamine 2000 (Invitrogen). The viral containing supernatants were collected 48-72 hours after transfection, infected into prostate cancer cells (PC-3 and DU145) and selected for stable gene insertion under 2 μg/ml puromycin.

### Statistical analysis

A paired t- test was used for statistically analyzing the data, and s5 significance is defined as P<0.05.

(Details materials and methods in supplementary text)

## SUPPLEMENTARY EXPERIMENTAL PROCEDURES AND FIGURES


