# Supplementary material for: E-selectin ligand-1 controls circulating prostate cancer cell rolling/adhesion and metastasis
Source: Oncotarget. 2014 Oct 6;5(23):12097–110. doi: 10.18632/oncotarget.2503 (PMC4322988; doi:10.18632/oncotarget.2503)
Supplement: Supplementary file 1 [file oncotarget-05-12097-s001.pdf]

SUPPLEMENTARY EXPERIMENTAL PROCEDURES AND FIGURES

Cell lines and cultures

$PHEC_T$ and $PHEC_{M,}$ LNCaP-P, LNCaP-R, CWR22R-1, CWR22R-2, PC-3, and DU145 cell lines were cultured with RPMI-1640 medium (GIBCO) containing 10% fetal bovine serum and 1% streptomycin/ penicillin. Primary Human Umbilical Vein Endothelial Cells (HUVEC) were maintained in Medium 200 (Gibco, Carlsbad, CA), containing 1x low LSGS and 2% heat-inactivated fetal bovine serum.

Western blot analysis

Whole cell lysate and tissue lysate were prepared following standard protocol [1]. Approximately 10 µg/lane of protein was loaded in 10% and 12% polyacrylamide gel with Tris/Glycine running buffer system. Antibodies were used according to manufacturer recommended dilution (from 1:100 to 1:5000) and HRP-conjugated secondary antibody was used to 1:2000 to 1:10,000 dilutions. Antibodies were used for western blot are: from Cell Signaling: MEK (Cat # 4694s), phospho MEK (Cat # 2338s), ERK (Cat # 4376), phospho ERK (Cat # 4376); ABCam: ESL-1 (Cat # ab103439); Senta Cruz: nRAS (Cat # Sc-31), cFOS (Cat # sc-52), and GAPDH Cat # sc-32233).

Invasion assay

Invasive activity was determined by using BioCoat Matrigel Invasion Chamber (BD Bioscience) according to the protocol recommended by the manufacturer. 5×10^4 cells/well were plated onto an 8 µm pour sized 24 well chamber of BioCoat Matrigel Invasion Chamber. Cells were suspended in serum free cell culture medium on the top chamber where a matrigel coated filter separates the top from the bottom chamber. The bottom chamber contained cell culture media with 10% fetal bovine serum as chemoattractant. After incubation for 24 hours at 37°C and 5% CO_2 atmosphere, the non invasive cells were removed with a cotton swab. The cells that had migrated through the membrane and stuck to the lower surface of the membrane were fixed and stained with 1% Toluidine Blue suspended in 1% sodium borate solution [2]. For quantification, cells were counted using an inverted light microscope within fields at a magnification of 50X.

Hematoxylin and eosin (H&E) staining

Mice tissues were fixed with 10% formalin and embedded with paraffin using Tissue-Tek Tissue Embedding Console System. Formalin-fixed and paraffin-embedded tissues were sliced (5 µm thick) using Accu-Cut SRM 200 Rotary Microtome. H&E staining was performed using Hematoxylin (Ricca Chemical Company Catalogue No. 3530-16) and Eosin (Eosin Y, 1% Alcoholic Solution, EMD, Catalog no. 588X-75) following the standard company protocol.

mRNA extraction and conversion to cDNA

Cells were lysated using TRIZOL reagent (Invitrogen) and RNA was extracted following the recommended protocol and converted to cDNA by C1000 Thermal Cycler PCR machine (Bio-Rad) by reverse transcriptase polymerase chain reaction (RT-PCR) following manufacturer's (Invitrogen) protocol.

Semiquantitative RT-PCR and real-time PCR assay

Total RNA was extracted from cells using Trizol (Invitrogen) following the company protocol; extracted RNA was measured by Nanodrop 2000c spectrophotometer and converted to cDNA by RT-PCR with reverse transcription by the iScript reverse transcriptase kit (Bio-Rad) using C1000 Thermal Cycler (Bio-Rad) following the protocol: 25 for 300s, 42 for 1800s, 85 for 300s 4 for 10s n. Real-time PCR was done with SYBR Green PCR Master Mix on an C1000 Thermal Cycler CFX96 multicolor real-time PCR detection system (Bio-Rad) following protocol: 50°C for 2 min, 95°C for 10 min then 40 cycles of denaturation at 95°C for 15 sec, annealing at 60°C for 1 min and extension at 55°C for 10 sec and 95°C for 5 sec [3]. Primers were purchased from. Sequences of primers are followings:

PSCA 5′- TGCTTGCCCTGTTGATGGCAG -3′(forward)
5′- ACGTGAGCCGGACGACGAGAC -3′(reverse)
CK-5 5′- TGGTCTCCCGTGCCGCAGTTCTAT -3′(forward)
5′- ATTTGGGATTGGGGTGGGGATTCT -3′(reverse)
CD-133 5′- TCTTGACCGACTGAGACCCAAC -3′(forward)
5′- ACTTGATGGATGC ACCAAGCAC -3′(reverse)
NANOG 5′- TTCAGTCTGGACACTGGCTG -3′(forward)
5′- CTCGCTGATTAGGCTCCAAC -3′(reverse)
MMP-3 5′- TGGCATTCAGTCCCTCTATGG -3′(forward)
5′- AGGACAAAGCAGGATCACAGTT -3′(reverse)

IGF-1 5′- ATGCTCTTCAGTTCGTGTGTG -3′(forward)
5′- GCACTCCCTCTACTTGCGTTC -3′(reverse)
TRMP-1 5′- ATCCGTGTATCCTATGACACCA -3′(forward)
5′- CATCCCCTACGTGGCTGATAA -3′(reverse)
SMAD-4 5′- GTCTGAGCATTGTGCATAGTTTG -3′(forward)
5′- GACGGGCATAGATCACATGAG -3′(reverse)
RORB 5′- CTTGGGCAGGAATAAGAAGCAT -3′(forward)
5′- CTTGGGCAGGAATAAGAAGCAT -3′(reverse)
PSGL-1 5′- TGTTGCTGATCCTACTGGGC -3′(forward)
5′- CTTGCCCAGGGACCACAACTC -3′(reverse)
ESL-1 5′- ATGTGGCAGTATTCGGCTTGG -3′(forward)
5′- CACAGTGGTAGACTCAGGGGT -3′(reverse)
CD44 5′- CTGCCGCTTTGCAGGTGTA -3′(forward)
5′- CATTGTGGGCAAGGTGCTATT -3′(reverse)
GAPDH 5′-CCACATCGCTCAGACACCAT- 3′(forward)
5′-ACCAGGCGCCCAATACG-3' (reverse)

Cell proliferation assay (MTT assay)

Cells were seeded in 24-well tissue culture plates (3 wells for each sample; triplicate) at a density of 1×10^3 to 5×10^3 cells/cm^2 (according to cell type) in RPMI-1640 cell culture media containing 10% FBS. At day 0, 2, 4 and 6 time points, the medium was replenished and cell proliferation was determined by 3-[4,5-dimethylthiazol-2-yl]-2,5-diphenyltetrazolium bromide (MTT) (Sigma). Stock solution of MTT (5 mg/mL PBS) was added into each well to a 10-fold dilution. After 2-hour of incubation at 37°C, the DMSO solution was added to extract the formazan product and the absorbance was recorded [1].

Establishment of shESL-1 PCa cells

Sequences are following:
shESL-1 #4: AAACTTGTAAGAAAACCGA (sense)
shESL-1 #5: AAACTTGTAAGAAAACCGA (sense)
scESL-1: ATCTCGCTTGGGCGAGAGTAAG (sense)

SUPPLEMENTAL REFERENCES

1. Cai Y, Lee YF, Li G, Liu S, Bao BY, Huang J, Hsu CL, and Chang C. A new prostate cancer therapeutic approach: combination of androgen ablation with COX-2 inhibitor. International journal of cancer Journal international du cancer. 2008; 123(1):195–201.
2. Ting HJ, Bao BY, Reeder JE, Messing EM and Lee YF. Increased expression of corepressors in aggressive androgen-independent prostate cancer cells results in loss of 1alpha,25-dihydroxyvitamin D3 responsiveness. Mol Cancer Res. 2007; 5(9):967–980.
3. Bao BY, Ting HJ, Hsu JW, Yasmin-Karim S, Messing E and Lee YF. Down-regulation of NF-kappaB signals is involved in loss of 1alpha,25-dihydroxyvitamin D3 responsiveness. J Steroid Biochem Mol Biol. 2010; 120(1):11–21.

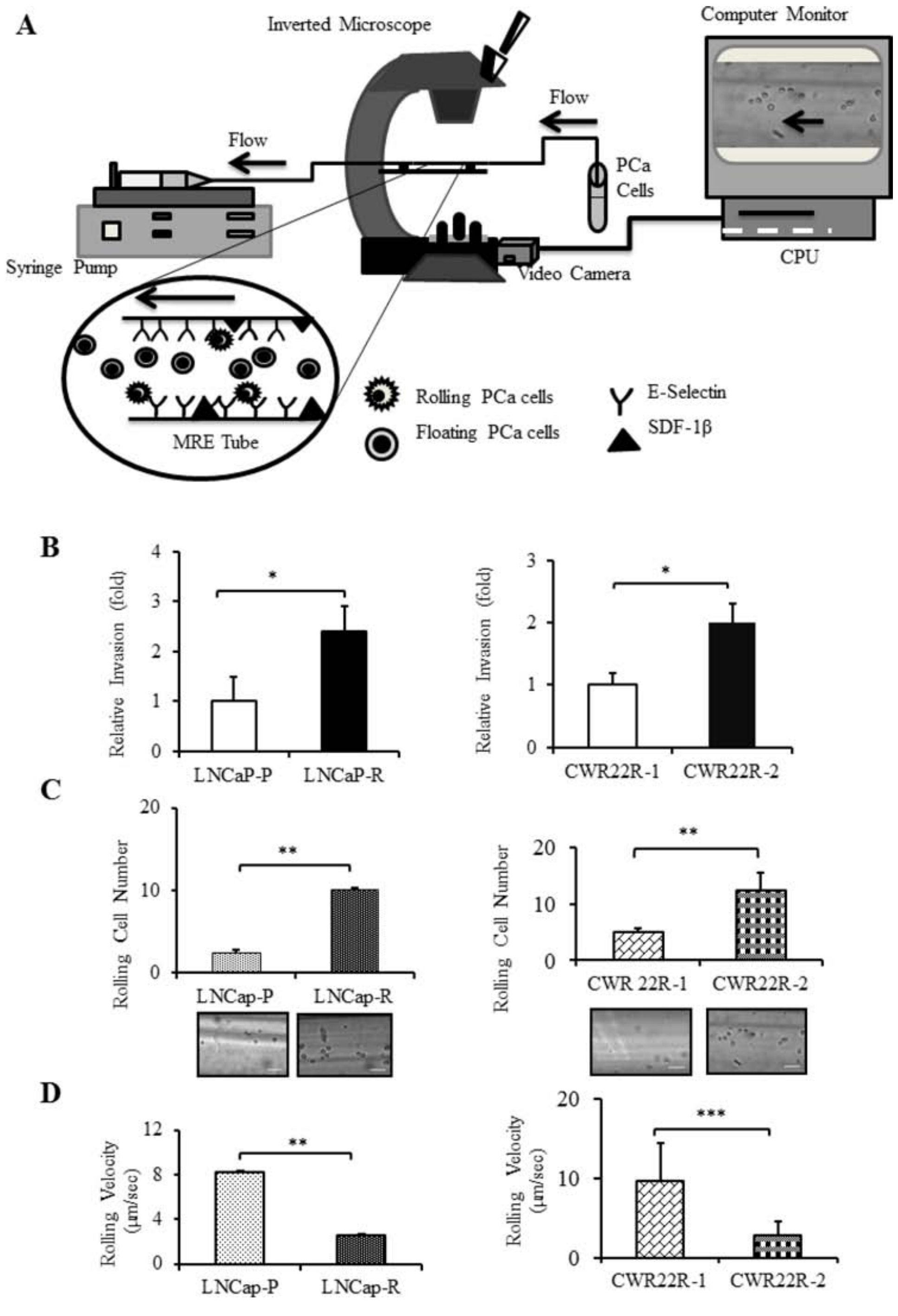

Supplementary Figure S1: Setting up a fluid dynamic model to study CTCs rolling behavior and comparing LNCaP sublines: LNCaP-P and LNCaP-R and CWR22R sublines: CWR22R-1 and CWR22R-2 PCa cell lines. **(A)** Diagrammatic representation of the dynamic microchannel system. **(B)** Comparing the aggressiveness by an invasion assay. **(C)** Comparing the rolling cell number and **(D)** rolling cell velocity. Scale bar represents 50 μm. Error bars indicate SEM. *, $P < 0.05$; **, $P < 0.01$; ***, $P < 0.001$.

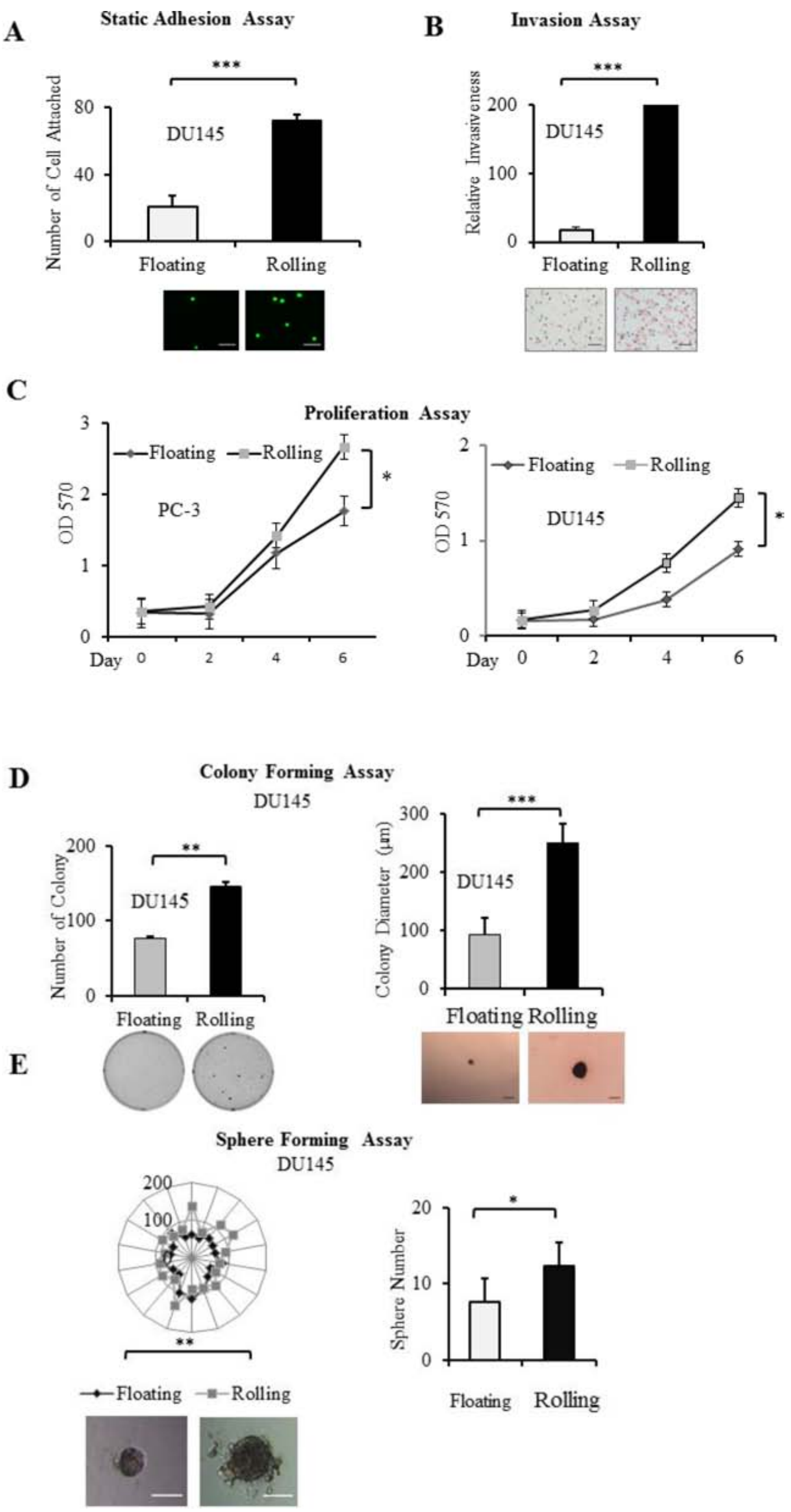

Supplementary Figure S2: Sorted rolling DU145 PCa cells demonstrate higher aggressive phenotype and stemness. **(A)** static adhesion assay. **(B)** relative invasiveness **(C)** proliferation assay **(D)** soft agar colony formation assay. **(E)** 3D sphere forming assay. Radar graph shows diameter and bar graph represents sphere number. Error bars indicate SEM. Scale bar represents 50 µm. Error bars indicate SEM. *, $P < 0.05$; **, $P < 0.01$; ***, $P < 0.001$.

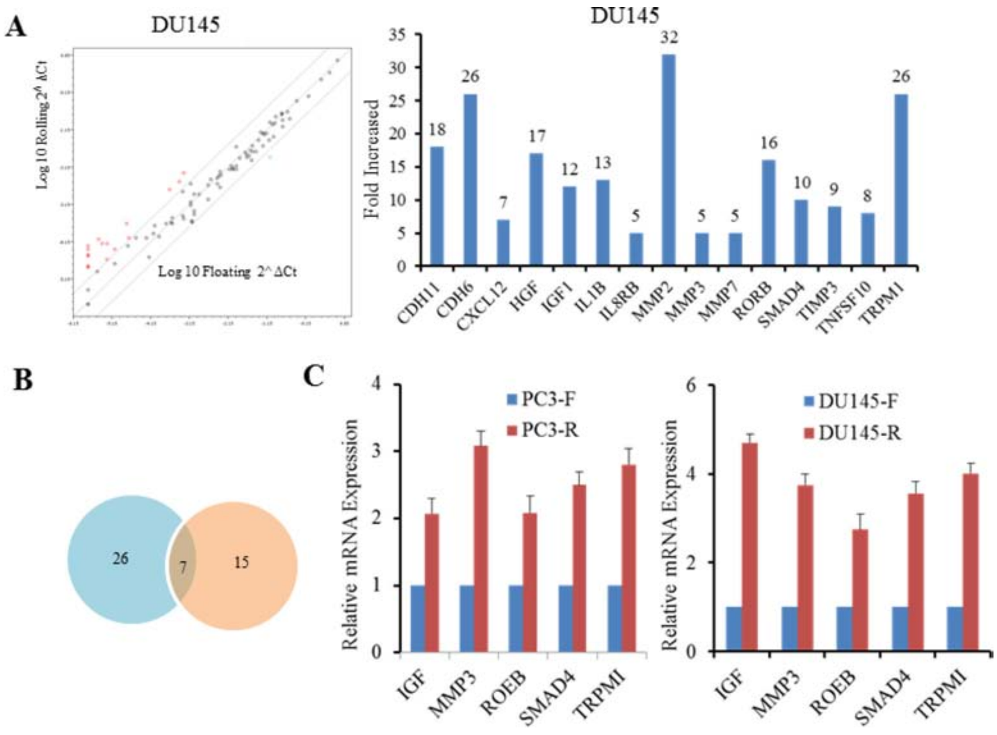

Supplementary Figure S3: Human metastatic gene array. (A) Scatter plot analysis displays of the up- and down-regulated in sorted DU145 cells. **(B)** Differential displayed genes compared between rolling *vs.* floating PC-3 and DU145 shown in a Venn diagram. **(C)** Q-PCR of some of the common genes. Error bars indicate SEM.

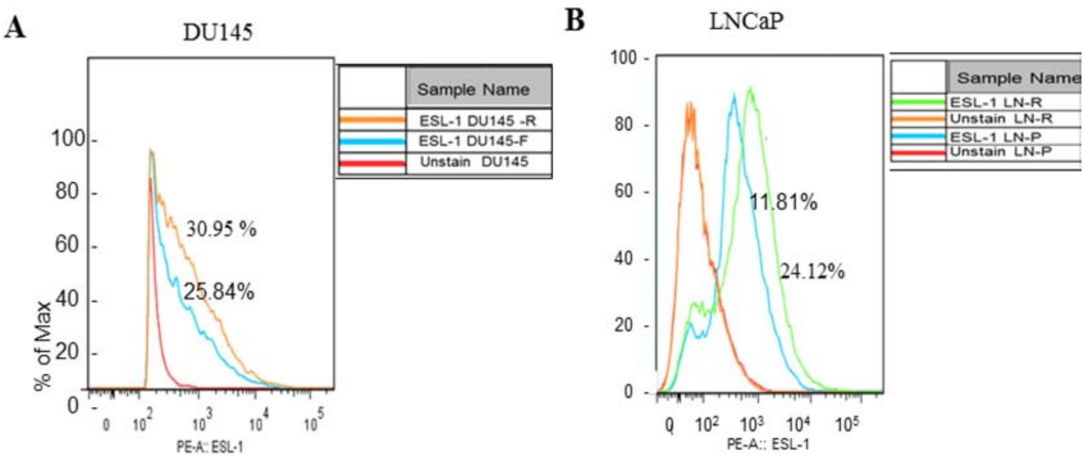

Supplementary Figure S4: ESL-1 was elevated in rolling PCa cells. (A) Flowcytometry data showing surface expression of ESL-1 in sorted rolling and floating DU145 PCa cells. **(B)** Flowcytometry assay of the total ESL-1 level in LNCaP sublines. All experiments were performed at least two times.

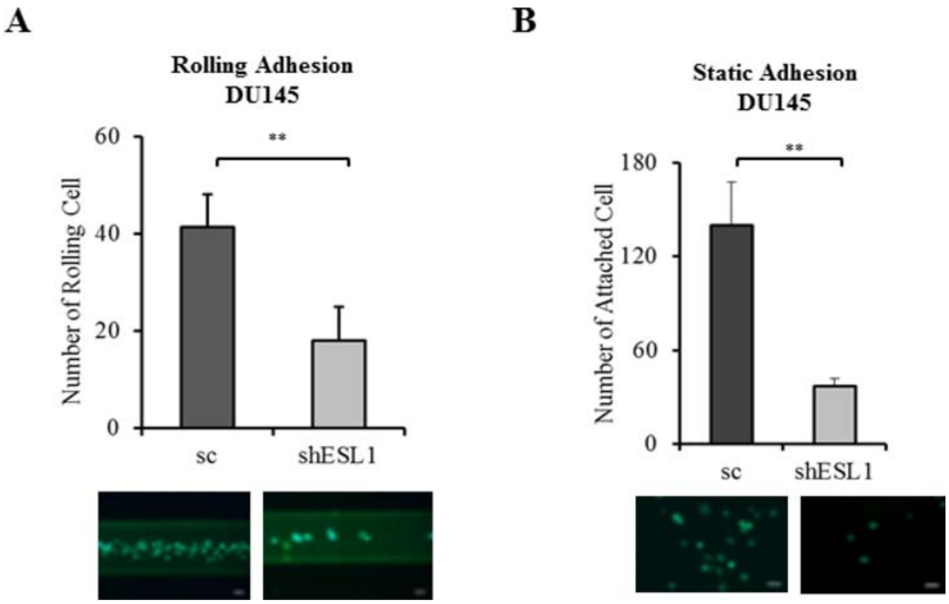

Supplementary Figure S5: ESL-1 is responsible for scESL-1 and shESL-1 DU145 cell PCa rolling and aggressiveness. (**A**) average rolling/adhesion cell number under flow of shear stress 1 dyne/cm^2. **(B)** average adherent cell numbers on HUVEC cells. Scale bar represents 100 µm. Error bars indicate SEM. **, P < 0.01.

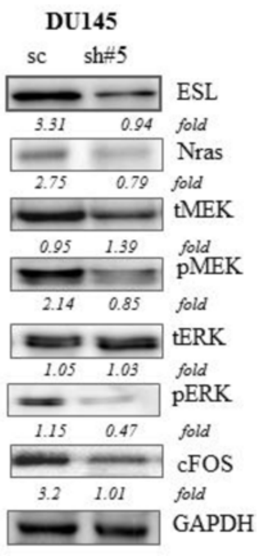

Supplementary Figure S6: ESL-1 activates RAS-MAP kinase signaling pathway. Western blot analysis of key molecules of RAS-MAP kinase signaling pathway from cell lyste of shESL-1 and scESL-1 of DU145 cells. GAPDH was used as a housekeeping gene.

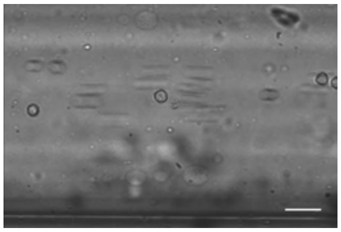

Supplementary Movie 1: Video of PC-3 PCa cells rolling on E-selectin/SDF-1β coated microtubes under wall shear stress 1 dyne/cm^2. Video clip was captured using an Olympus IX-81 microscope at 200x with phase contrast and a rate of 30frames/second. Scale Bar represents 50μm.
